# Critical Human and Organizational Factors for Structural Safety in the Dutch Construction Industry

**DOI:** 10.1002/ajim.23681

**Published:** 2024-12-03

**Authors:** Xin Ren, Karel C. Terwel, Ming Yang, Pieter H. A. J. M. van Gelder

**Affiliations:** ^1^ Safety and Security Science Group Faculty of Technology, Policy and Management, Delft University of Technology Delft The Netherlands; ^2^ Structural Design and Building Engineering Faculty of Civil Engineering and Geosciences, Delft University of Technology Delft The Netherlands

**Keywords:** human and organizational factors, human error, structural safety, survey, the HOPE framework

## Abstract

**Background:**

Human errors are widely acknowledged as the primary cause of structural failures in the construction industry. Research has found that such errors arise from the situation created by human factors and organizational factors embedded in the task context. However, these contextual factors have not been adequately addressed in the construction industry. Therefore, this study aims to identify the critical Human and Organizational Factors (HOFs) that influence structural safety in frequently performed tasks in structural design and construction.

**Methods:**

Through a comprehensive literature review, a framework consisting of potential critical factors called the HOPE framework, is presented. To identify the most critical HOFs that contribute to human error occurrences, a questionnaire survey to experts in the Dutch construction industry was conducted. Finally, the resulting framework was compared with three actual structural failures for validation.

**Results:**

This study shows that the HOFs should be extended with project‐related factors (P) and working environment‐related factors (E) due to the fact that these task contextual conditions play a significant role in shaping professionals' on‐the‐job performance. Furthermore, a survey identified 14 HOFs as critical in contributing to an error‐prone situation in the structural design and construction tasks.

**Conclusion:**

The presented HOPE framework and the identified critical HOFs for structural safety can assist engineers with better hazard identification and quality assurance in practice.

## 1 Introduction

The construction industry is one of the most unsafe industries worldwide [1]. It witnessed the highest number of fatalities among all industries in the United States in 2021 [2] and consistently records the largest amount of work‐related fatal injuries in the United Kingdom [3]. A large proportion of fatal injuries in the construction industry are caused by structural failures and collapsing objects [3]. For example, the collapse of a five‐story apartment building in Cairo, Egypt, on July 17, 2023, claimed 13 lives. Besides, a railway bridge collapsed under construction in Mizoram, India, on August 23, 2023, killing at least 26 construction workers. As can be seen, structural failures can result in enormous detrimental social and individual consequences, such as financial losses, reputation losses, and even injuries and fatalities. Therefore, the safety of structures is critical to the safety of structural users and construction workers. Achieving and maintaining a safe state, or an expected quality state of the constructed structure is one of the primary goals in the construction industry. To meet this fundamental requirement, unacceptable structural failures, such as (partial) collapse and structural damage that can lead to the loss of structural integrity [4], should be avoided.

### 1.1 Causes of Structural Failures

What causes structural failures? Many studies and accident investigations exist on identifying the root causes of structural failures [5, 6, 7, 8, 9, 10]. Numerous studies have identified the primary cause of structural failures and near‐miss cases as human errors rather than technical issues [4, 6, 11, 12, 13, 14, 15]. Therefore, human error is recognized as an essential issue to be tackled to achieve structural safety. As a result, a great amount of research efforts have been made to study various errors that play significant roles in affecting structural safety. For example, Walker pointed out that the error in defining the loads in design is the dominant error type (61%). Moreover, ignoring loads, ignoring structural behavior, mistakes in calculations and drawings, and inadequate instructions are recognized as the primary errors contributing to structural failures [6]. After studying 604 structural and construction failures from 1975 to 1986 in the United States, Eldukair and Ayyub concluded that construction errors, among errors in the plan, design, and utilization phase, are the highest contributing causes for structural defects and failures [8].

### 1.2 Human and Organizational Factors (HOFs)

**Figure 1:**
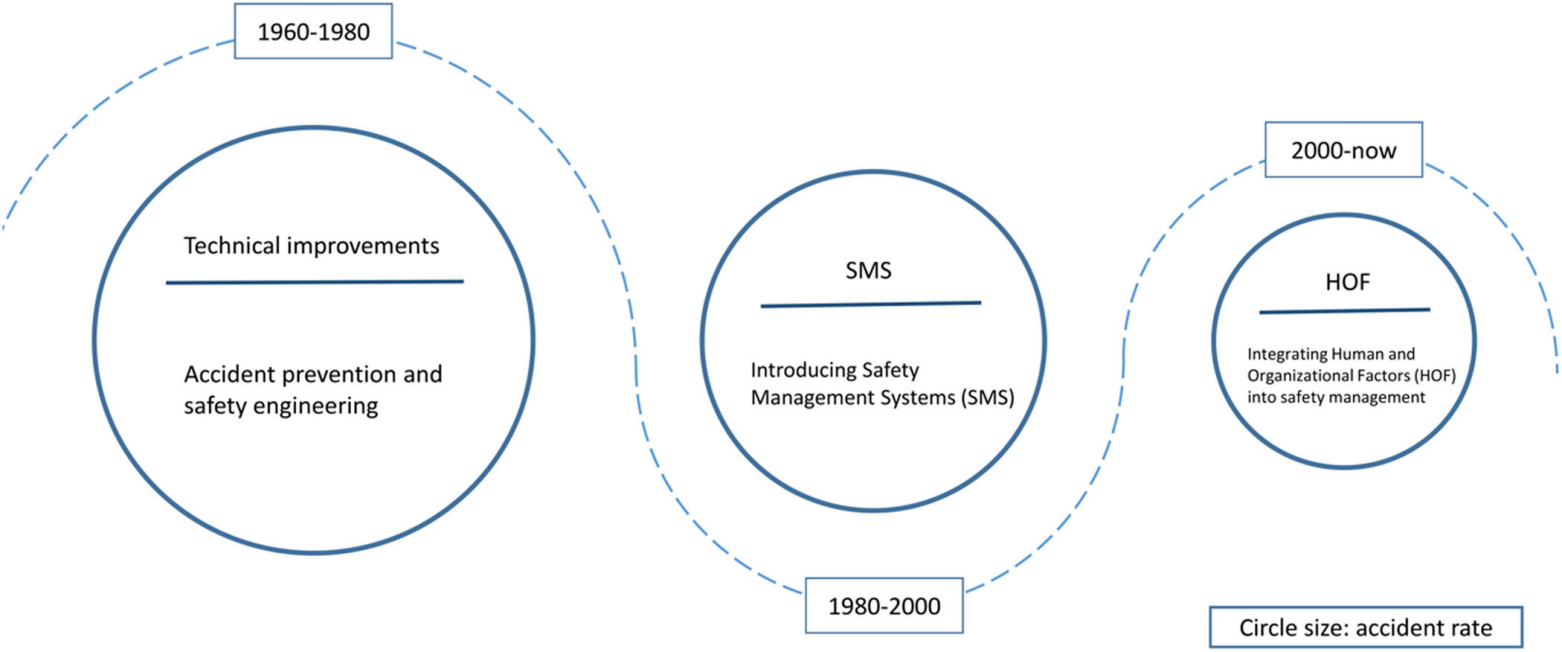
The development of approaches toward industrial safety. Figure adapted from Daniellou et al. [18]

However, the latest development in safety science no longer views human error as the root cause for accidents, but rather as the symptom of troubles that are deeply embedded in or at the higher hierarchy of the system [16]. Human errors arise from these unfavorable system conditions and work contexts, specifically, how the system is designed and managed, and the way humans interact with the system [17]. These underlying factors can shape the performance of people at work and potentially lead to the occurrence of human errors and accidents. They include human performance‐related factors, such as physical and mental conditions of the personnel at the job, and organization‐related factors that concern the organizational process and management strategies, which are termed the HOFs. Figure 1 illustrates the progressive development of industrial safety approaches. It is clear that the approaches toward industrial safety evolved from focusing on the technical aspects to improve safety to introducing the Safety Management Systems (SMS) to account for the management facet of the overall system safety, and the latest to take the human and organizational perspectives of the system into consideration. The HOF concept arose after the widely accepted man‐made disasters [19] and the normal accidents theory [20] of accident causation, representing a system approach toward human error [17]. Consequently, a better solution toward the human error issue lies in the enhanced understanding of the HOFs.

In the construction domain, human errors are still frequently viewed as the root cause of structural failures. The underlying HOFs behind the errors are very often neglected. As a result, the factors that contribute to the error occurrence can repeatedly cause trouble. Blockley provided the foresight that civil engineering failures are as much of a human and organizational phenomenon as a technical failure [21]. Moreover, Elms specified that it is important to be aware of the factors that lead to increased error proneness when handling structural safety [22]. Therefore, HOFs are key to treating human errors and making progress in improving structural safety. As pointed out by Melchers, human error and human intervention have not been studied extensively in the structural reliability field [23]. The current research into the contributing HOFs in structural failures is far from adequate. Thus, a better understanding of the HOFs associated with structural safety is in demand.

Hence, this study aims to contribute to the knowledge of HOFs by identifying critical task‐specific HOFs that can lead to the occurrence of human error in the structural design and construction process.

## 2 Materials and Methods

**Figure 2:**
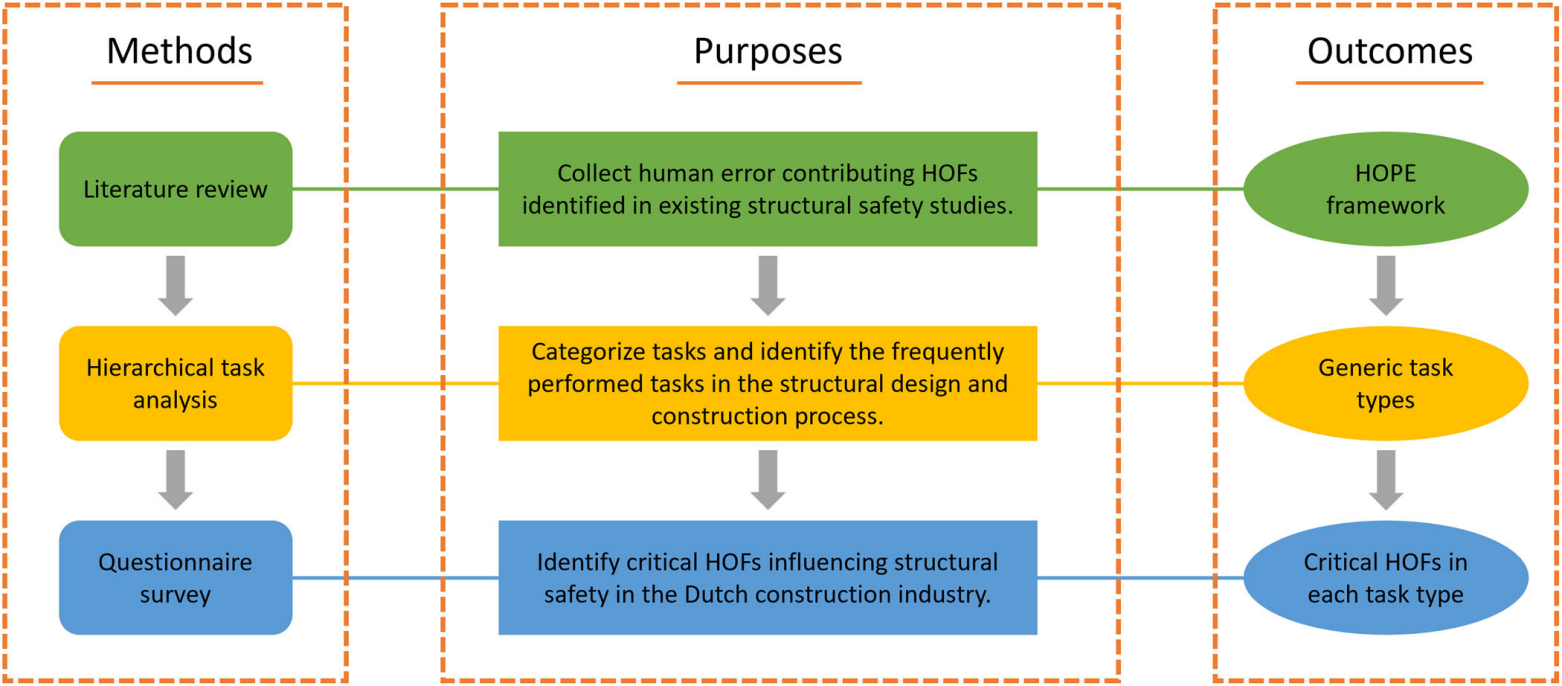
The research workflow.

To reach the above research goal, several methods were applied in this study and some results were obtained consequently. The overall research workflow of this study is outlined in Figure 2.

### 2.1 Literature Review

Important factors that affect structural safety, which have been identified in existing studies were collected from a comprehensive literature review [24]. As a result, a hierarchical HOFs framework is proposed. Moreover, the definitions of each factor and the distinguished task types in structural design and construction, termed as the Generic Task Types (GTTs), are provided. Furthermore, the critical factors of each GTT were identified through a survey to experts in the Dutch construction industry.

### 2.2 Questionnaire Survey

**Figure 3:**
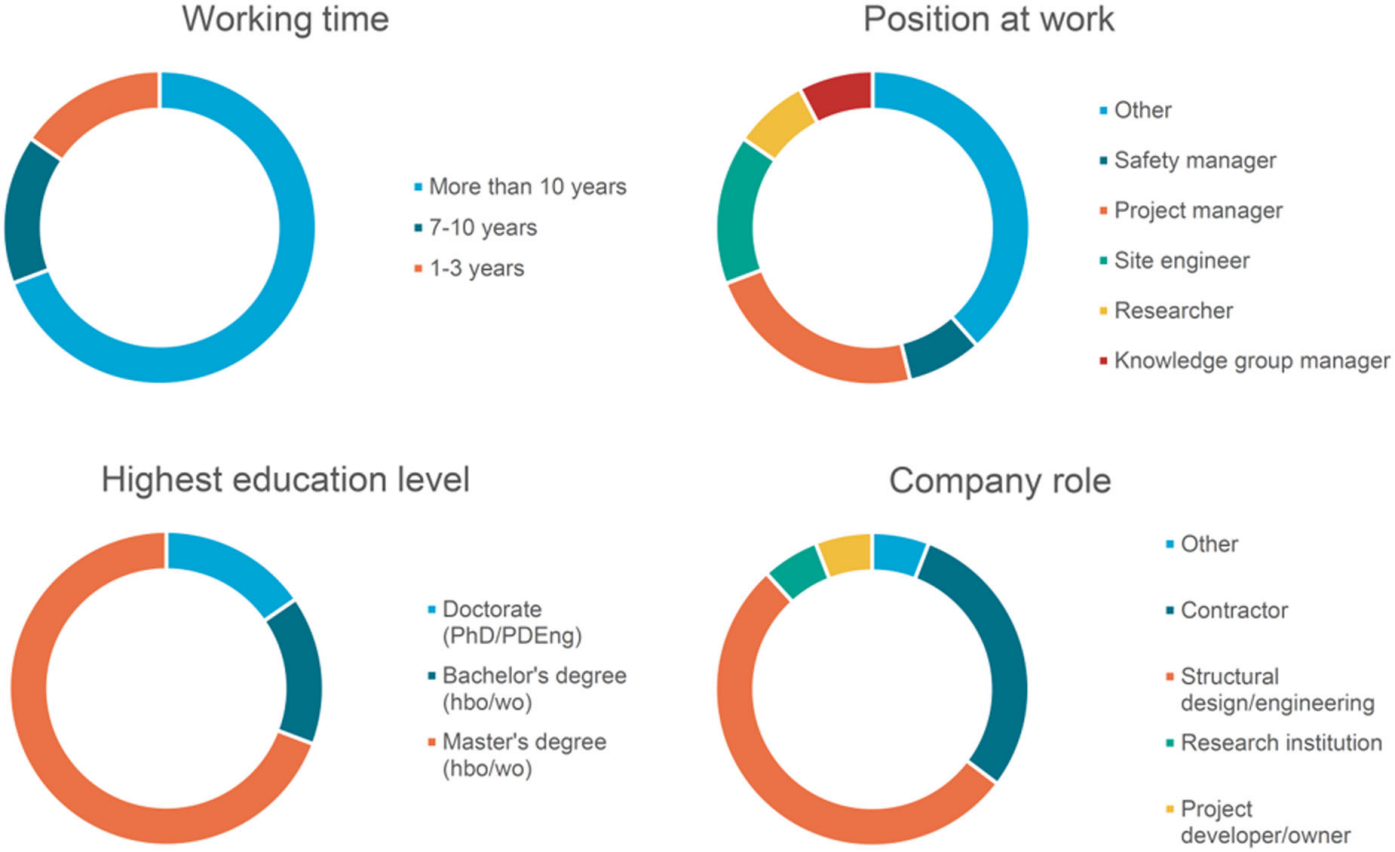
Profiles of the survey responding experts.

To identify the most critical HOFs that contribute to human error occurrences and consequently influence structural safety in the Dutch construction industry, a questionnaire survey was designed and issued to experts in the Dutch construction sector. For practicability considerations, the subcategory HOFs were used in the survey study instead of the specific HOFs. The questionnaire is designed such that each question inquiries about the critical HOFs for a specific GTT. Using a 5‐point Likert scale, the respondents were asked to rate each factor on how influential it is on the type of task under consideration (i.e., not‐at‐all influential, slightly influential, somewhat influential, very influential, extremely influential). An example question from this questionnaire can be seen in the Figure S‐1. The profiles of the responding experts are shown in Figure 3.

### 2.3 Analytic Hierarchy Process (AHP)

The collected expert judgment data were subsequently analyzed using the AHP to elicit a rational consensus concerning the relative importance ranking of the HOFs for each GTT. AHP is a widely used method in solving Multi‐Criterion Decision Making (MCDM) problems. It is a pair‐wise comparison method which provides mathematical assessments to prioritize decision criteria and alternative options. Based on rational judgments, it assigns distinct weights to the alternatives with regard to their contribution to the decision goal. AHP can derive both group and individual preferences. Developed by Prof. Saaty [25, 26], AHP has been applied in a wide range of domains, such as logistics, manufacturing, policy, and construction for various purposes such as planning, optimizing, risk analysis, and resource allocation [27]. In the construction field, AHP has been primarily applied for risk management, including risk identification and assessment, as well as risk‐informed decision‐making support [28]. For example, AHP is used to develop a framework for injury risk prioritization so that an adequate safety budget can be secured during the construction project planning phase [29]. In addition, AHP and the Failure Mode and Effect Analysis (FMEA) are combined with fuzzy logic to assess the criticality of potential risks in construction for better risk management [30].

## 3 Results

### 3.1 The Human‐Organization‐Project‐Environment (HOPE) Framework

Based on an extensive literature review on the topic of HOFs influencing structural safety [24], a comprehensive set of HOFs have been identified and analyzed. Consequently, a framework that consists of the widely acknowledged HOFs is proposed for proactive structural safety management in the construction industry. In this framework, the identified specific HOFs are further analyzed and summarized into 17 middle hierarchy subcategory HOFs. Some less frequently recognized specific HOFs are excluded and a few correlated subcategories are merged. Beyond this, these subcategory HOFs are classified into four main categories on the top hierarchy, which are the Human factors, the Organizational factors, the Project factors, and the Environmental factors. The project‐related factors and the working environment‐related factors are also included in this framework along with the human and organization‐related factors due to the fact that these task contextual conditions play a significant role in shaping professionals' on‐the‐job performance. As a result, a hierarchical HOPE framework is proposed. The final synthesized framework that embodies all three layers of factors is presented in Table 1.

**Table 1:** The HOPE framework.

Main category	HOFs	Specific HOFs
Human factors	Professional competence	Professional knowledge and skills
		Professional insights/anticipation
		Education
		Training
		Experience
	Trust	Over‐confident about traditional approaches and past experience
		Overly confident in engineering software and computer analysis
		Reliance on other parties
	Attitude	Motivation
		Commitment to the job
		Negligence and carelessness
		Violation of protocols, standards, and regulations to save effort
	Well‐being for duty	Physical health condition
		Mental health condition
		Fatigue
	Comprehensive abilities	Management skills
		Social‐communicative skills
		Teamwork skills
		Ability to learn
		Decision‐making ability
		Network and critical thinking
Organizational factors	Information flow	Communication quality
		Information availability and quality
		Information overload
	Task management	Task planning and preparation
		Task coordination and collaboration
		Change management
		Conflict management
	Organizational characteristics	Organization structure
		Organization stability
		Team size
		Responsibility division
		Support and provision from the parent company
	Quality assurance	Supervision
		Design checking
		Construction inspection
		Protocols/procedure/regulation availability and quality
	Risk analysis and management	Risk identification
		Risk analysis
		Risk and safety management
		Follow‐up warnings
	Engineering climate	Safety culture
		Structural safety goals
Project factors	Complexity	Task complexity
		Project type and size
		Concurrent working
		Many parties involved
	Stress	Time pressure
		Budget pressure
		Workload
	Fragmentation	High personnel rotation
		Task fragmentation
	New and unfamiliarity	New or advanced structures
		New technology or construction materials
		New or unfamiliar construction methods
Environment factors	Equipment	Correct equipment availability
		Equipment condition
		Ergonomics (human‐machine‐interface)
	Working conditions	Physical working environment
		Interpersonal/team environment
		Weather conditions
		Time of the day

#### 3.1.1 Definitions of HOFs

The HOFs are a similar concept as the Performance Shaping Factors (PSFs) or Performance Influencing Factors (PIFs), which are widely applied in the Human Reliability Analysis (HRA) domain. These factors are considered the contextual factors surrounding the task and influence the individual or team performance in completing the assigned task. HRA uses qualitative or quantitative methods to evaluate the human error occurrence potential by assessing the effects of PSFs or PIFs on task performance. Therefore, task‐specific PSFs are key for Human Error Probability (HEP) estimation. It is essential that these HOFs are clearly defined under the construction industry context so that confusion is avoided when applying them in task success or failure outcome evaluation and prediction. Thus, the subcategory HOFs in the HOPE framework are defined in Table 2.

**Table 2:** The definitions of HOFs.

Factor	Definition	Aspects of consideration
F1. Professional competence	The degree of utilization of professional knowledge, skills, and good judgment related to one's profession, which reflects one's readiness to work in a specialized area or profession.	Professional knowledge; professional skills; education; training; experience
F2. Trust	To have confidence over past experience or the work of teammates or other project participants; or blind belief in the results given by a computer program. Here trust refers to overconfidence or blind trust behavior which leads to careless examination or limited checking.	Overly confidence over past experience; over‐reliance on computer analysis/overly confidence in engineering software; blind trust/assuming errorless output from others; over‐confident about traditional approaches
F3. Attitude	Attitude shows the task performer's commitment toward the task at hand, whether they are willing to pay effort to achieve the task goal successfully.	Motivation; commitment; violations
F4. Well‐being for duty	Whether or not the task performer is physically and mentally capable of accomplishing the task successfully. For instance, fatigue, drug effects, and emotional instability might lead to errors while performing a task.	Mental resilience; physical resilience; fatigue
F5. Comprehensive abilities	Comprehensive abilities refer to the capabilities an individual possesses in addition to professional competence. These abilities include self‐management skills, teamwork and social‐communicative skills, ability to learn, critical thinking, network thinking and keeping an overview of the whole structure/project in mind while conducting divided task steps, and so forth.	Management skills; social‐communicative skills; ability to learn; critical thinking; network thinking
F6. Information flow	Information flow refers to the exchange of desired information between individuals and parties in the design and construction process. This consists of quality information being created, safely stored, and transferred to the targeted party on time so that a mutual understanding of the information is reached.	Communication; information availability and quality; information overload
F7. Task management	The planning, organizing, controlling, and coordinating of the task process and the task performers to achieve the task goal.	Teamwork; coordination; planning/preparation; change management
F8. Organizational characteristics	Organizational characteristics are aspects of organizations (e.g., structural engineering company, contractor, the whole project organization, etc.) that can be identified in relation to performance, such as the organization structure, team size, responsibility division, organization stability, and so forth.	Support and provision from parent company; organization structure; team size; responsibility division; organization stability
F9. Quality assurance	Quality assurance is part of quality management focused on fulfilling quality requirements of the structure via supervision, regulation, checking, and inspections.	Supervision; design checking; construction inspection; protocols/procedure/regulation
F10. Risk analysis and management	Risk analysis is the process of identifying potential hazards and evaluating the probability and consequence of corresponding failure and accidents. Risk management is to prioritize the risks and coordinate the available resources and measures to minimize the risk and prevent the occurrence of undesired events.	Hazard identification; risk analysis; risk management; follow‐up warnings
F11. Engineering climate	Engineering climate is the shared value, common attitude, collective goal, and group behavior shared toward structural safety and reliability by the majority of people within the workplace or project. It can be characterized as “the way we do things around here.”	Safety culture; safety goals
F12. Complexity	Complexity refers to how difficult the task is to perform in the given context. A complex task means it requires great mental efforts such as work related (short term) memory and knowledge to accomplish the task successfully.	Task complexity; project type and size
F13. Stress	Stress refers to the mental or emotional tension caused by constrained or undesirable conditions and circumstances at work, which will impede the task performer in completing a task. Stress can result from time pressure, budget constraints, and high workload due to limited staffing, and so forth.	Time pressure; budget pressure; workload
F14. Fragmentation	Fragmentation refers to the fact that the project is divided into small working packages that are assigned to highly specialized teams. This means that it requires great communication, coordination, and management efforts to accomplish the project successfully.	Frequent personnel change; lack of project overview and network thinking
F15. Equipment	The available equipment for conducting a task. The equipment includes hardware such as machines and tools and software like structural modeling and analysis programs. The influence of equipment on human error can be considered from the availability of desired equipment and their conditions, as well as Human‐Machine Interface (HMI, how the operator interacts with the equipment to correctly perform the task).	Equipment condition; ergonomics (HMI)
F16. Working conditions	Working conditions refer to the physical and interpersonal working environment at the workplace. It considers aspects such as weather conditions (rain, snow, wind, etc.), physical working environment (darkness, noise, dust, heat, small space, etc.) and interpersonal environment (peer pressure, competition, etc.).	Working environment; interpersonal/team environment; weather effects

#### 3.1.2 GTTs in Structural Design and Construction

It is found that most human errors occur during the structural design and construction process [4, 13, 31]. Thus this study focuses on the tasks in these two critical phases. Given that there are numerous detailed tasks involved in the structural design and construction process, some frequently performed typical tasks, summarized as the GTTs, were identified through a Hierarchical Task Analysis (HTA) in this study. An HTA outlines the primary tasks in a process and further breaks them down into detailed elementary actions. A GTT represents a typical type of task that shares similar system interactions, cognitive demands, and potential affecting factors [32]. GTTs should be clearly defined, mutually exclusive, and subject to the same sets of HOFs that post the same amount of impacts.

An HTA was performed to analyze tasks involved in a reinforced wide slab floor structure design and construction. As a result, 109 bottom hierarchy detailed tasks were obtained in this HTA. With a comparison to the decomposed tasks in another two studies [33, 34] and the critical cognitive activities in the Cognitive Reliability and Error Analysis Method (CREAM) [35], 14 frequently performed GTTs in the structural design and construction process have been abstracted. The definition of each GTT, the involved phase, and example tasks are outlined in Table 3.

**Table 3:** Generic task types in the structural design and construction process.

Task type	Phase	Definition	Task example
Diagnosis based on knowledge/experience/situation	Design/construction	Examine the current situation and make a decision based on professional judgment.	Listing all load combinations
Derive value	Design	To find out the desired parameter value from text, a table or a graph; or to obtain necessary information (e.g., material properties) from another party (e.g., architect, material supplier, etc.).	Looking up for the minimum reinforcement percentage from a table
Consult code	Design	To consult the Eurocode for the corresponding design requirements and calculation methods.	Determining consequence class based on building function
Mechanical schematization	Design	The process of analyzing and visualizing the supports and forces that apply to the structure using mechanical schemas.	Choosing the appropriate type of supports; schematizing a load distribution on a structural element
Calculation	Design	The process of producing a desired value using the known input value(s) and the mathematical relationships between the input and the desired value.	Calculating self‐weight; calculating moment resistance
Comparison/ranking	Design/construction	To examine or look for the differences between values or things. To place values or things in an order according to a certain criterion.	Checking minimum or maximum reinforcement percentages; comparing design variants
Interaction with design software	Design	To digitally model, visualize, or analyze the structure by inputting and adjusting parametric values using computational software.	Drawing floor plans and cross‐sections
Documenting the design and prepare specifications	Design	To record the structural design in documents and write down the detailed requirements and instructions for the structural construction.	Writing a structural design report
Follow instructions and act	Construction	Follow the given instructions (by site engineer or supervisor) to perform the task at hand accordingly.	Binding/welding mesh reinforcement; pouring concrete
Consult drawings and specifications	Construction	To obtain and interpret information about how to perform the task to realize the design from the design drawings and specifications.	Reading specifications about the type and the amount of reinforcement should be applied
Measurement	Construction	To survey the dimensions and features of a physical entity or a space.	Measuring the center‐to‐center distance between two adjacent reinforcement bars
Interaction with hardware equipment (tools/machine)	Construction	Human cooperation with a machine or utilization of a tool.	Configuring a pre‐stressing bench (in a factory)
Communication	Design/construction	To share information with others via speaking, signals, documents, or other forms of communication.	Supplying the structural design to contractor
Checking/inspection	Design/construction	To carefully examine the designed or constructed structure to determine its accuracy, quality, or condition, or to detect the presence of errors or the lack of required elements.	Check by supervisor; structure inspected by specialist before delivery

### 3.2 Survey Results

#### 3.2.1 Application of AHP

**Figure 4:**
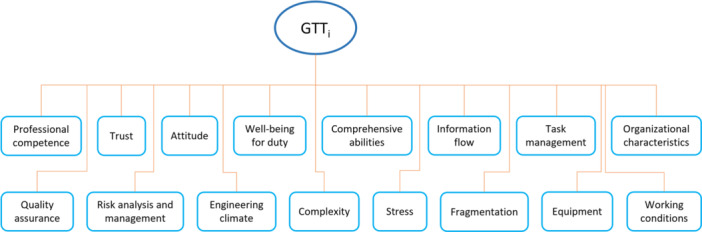
The GTT‐HOFs hierarchy.

We applied AHP to prioritize the criticality of the HOFs in each GTT. The established hierarchical structure is shown in Figure 4. It can be seen that for each GTT, there are 16 potential critical HOFs involved. However, there are only two levels in this hierarchy because the goal of our study is to identify the critical HOFs (as the criteria layer) without the HOFs management strategies (as the alternative option layer). Additionally, the questions in the questionnaire are designed for experts to rate the level of influence each factor has on the specified task type. In this way, the consistency ratio is always equal to 0 and thus the expert judgment consistency is guaranteed. Given the lowest level of influence (not‐at‐all influential) is numerically translated into 1, and the highest level of influence (extremely influential) can be numerically translated into 5, the current paired comparison ratings range from 1 to 5. Thus the ratings above 5 from the nine‐point scale in AHP [26] were not used in the formulated comparison matrix in this study. Since the final aim is to obtain the relative importance of these factors, the incomplete adoption of the nine‐point scale is believed to cause no concern to the final factors' relative importance ranking. To solve the current group decision problem, the geometric mean of all experts' ratings on one factor for one task type was first computed. It should be noted that the experts were equally weighted. Then these geometric mean results were used to formulate the pair‐wise comparison matrices. These decision matrices were subsequently solved to calculate the maximum eigenvalue and the corresponding eigenvector. Consequently, the normalized weight of each factor for each task type, which can be interpreted as the criticality level of these HOFs for each GTT, was obtained.

#### 3.2.2 HOFs' Weights Overview

**Figure 5:**
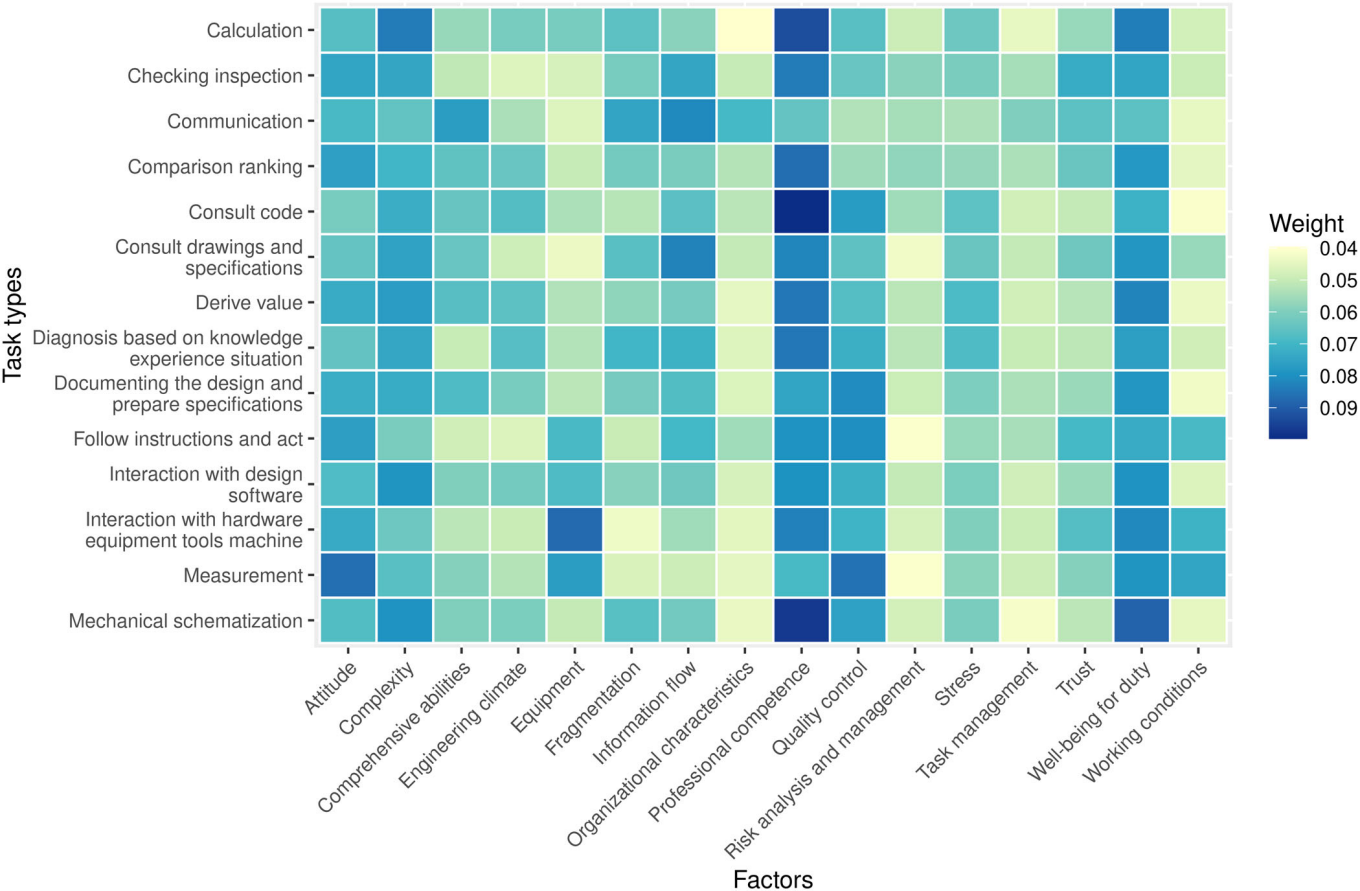
HOFs' weights overview. In this figure, the HOFs are outlined on the *x*‐axis and the GTTs are listed on the *y*‐axis. Therefore, each grid in this heatmap represents one factor in one task type. The color of the grid indicates the factor's weight, which reflects the factor's level of influence on this type of task. The factor with a higher weight is displayed with a darker‐colored grid.

The factor's normalized weight in each type of task has been calculated from the questionnaire survey data through AHP. The HOFs' weights range from 0.0397 to 0.0997. The arithmetic mean of these factors' weights is 0.0625. A matrix showing the normalized weights overview of each factor for each task type is presented as a heatmap in Figure 5.

It can be seen from Figure 5 that *professional competence* holds the highest weight among all HOFs, which indicates its significant influence on human performance in most of the GTTs. It is ranked by the experts as the most influential factor for eight task types, among which it is considered especially critical for task types of *consulting code*, *mechanical schematization*, and *calculation*. Apart from *professional competence*, *well‐being for duty* and *complexity* are also recognized as critical factors for the majority of GTTs. Both *well‐being for duty* and *complexity* are more influential on *mechanical schematization* and *calculation* type of task. Moreover, *attitude* and *quality assurance* are also selected as critical considerations for many GTTs, especially for *measurement* tasks.

On the other hand, *task management*, *organizational characteristics*, *risk analysis and management*, and *working conditions* are rated with lower weights in most GTTs. The reason for this low influence grading might lie in that these factors have a rather general, sometimes abstract nature when evaluating their influence for a specific error condition. In addition, *risk analysis and management* is mostly in the project planning phase rather than the design and construction phase. *Organizational characteristics* is a factor located in the upper stream of the project system, so its impact on task performance is indirect and, thus, difficult for the experts to make a judgment of its direct contribution to human error occurrence in these GTTs. Intuitively, *task management* should be an important factor with regard to task performance. Its low weight might be the result of the belief that individual errors can better be handled by quality assurance measures rather than management strategies of specific tasks. Another observation is that *working conditions* is considered less influential for GTTs related to structural design but more impactful for construction tasks. The reason behind this finding might be that structural design tasks are indoor office work whose working conditions are more favorable and reliable; whilst construction tasks on site are often outdoor, the working conditions are complicated and less favorable and controllable.

#### 3.2.3 Task‐Based Critical HOFs

**Figure 6:**
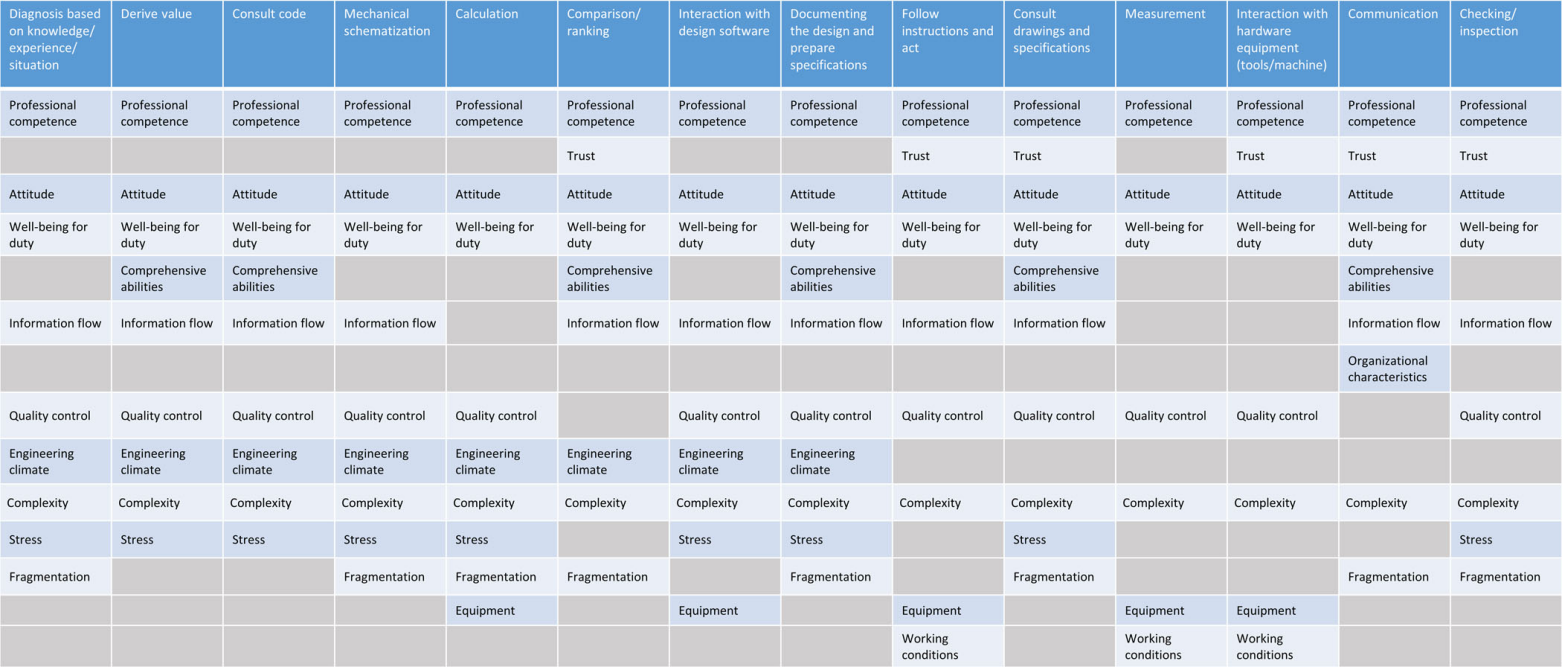
Critical HOFs in each task type.

Based on the calculated factor weights, considering the arithmetic mean of all factors' weights (0.0625), the factors with a weight above 0.06 (above average) are included as critical factors for a GTT. An overview of the critical HOFs for each GTT is outlined in Figure 6. It can be clearly seen that *professional competence*, *attitude*, *well‐being for duty*, and *complexity* are identified as critical factors for all 14 GTTs. The other widely recognized influential HOFs are *quality assurance* and *information flow*, which are considered critical in 12 GTTs and 11 GTTs, respectively. However, *organizational characteristic* is only considered influential for the *communication* task type. Moreover, *working conditions* is considered influential on human performance in three GTTs in the construction process. It is worth mentioning that the normalized weights of the factors *task management* and *risk analysis and management* are always below 0.06. Thus these two factors are not included in any GTTs as critical influential factors. Consequently, the critical HOFs set is left with 14 factors.

Another observation lies in the more vulnerable or robust task types. It can be seen from Figure 6 that most of the GTTs contain nine critical HOFs. With 10 influential HOFs, *documenting the design and preparing specifications* in the design process and *consult drawings and specifications* in the construction process are considered more vulnerable with regard to human error proneness since they contain more error‐inducing conditions. On the other hand, there are seven and eight critical HOFs included in the *measurement* and *interaction with hardware equipment (tools/machine)* task types in the construction process, which make them more robust against human errors.

### 3.3 Validation by Cross‐Comparison Against Actual Structural Failures

The Dutch Safety Board (DSB) is an independent body that investigates incidents and safety problems in a broad range of industries in the Netherlands. Until now, it has published accident investigation reports on three high social impact structural failure incidents in the Netherlands, including the temporary structure collapse of Rotterdam B‐tower in 2010 [36], the stadium roof collapse of FC Twente in 2011 [37], and the Eindhoven parking building floor collapse in 2017 [38]. A review of these reports confirmed the identified critical HOFs in this study and showcased the effectiveness of the proposed HOPE framework in guiding qualitative risk analysis for structural safety management.

#### 3.3.1 Rotterdam B‐Tower

On October 21, 2010, the third floor of the Rotterdam B‐Tower collapsed during the concrete casting process, resulting in severe injuries to five construction workers. Subsequent investigation of this accident identified the immediate cause as the instability of the temporary scaffolding support structure, which proved incapable of bearing the load of the poured concrete. Furthermore, four key underlying factors contributing to this failure were identified during the investigation. First, the personnel responsible for scaffolding construction lacked adequate training and supervision, aligning with the factors of *professional competence* (training) and *quality assurance* (supervision) within the HOPE framework. Secondly, the scaffolding was inspected prior to the concrete pouring. However, the identified load‐bearing capacity issue was not treated properly. This relates to the risk analysis and management (follow‐up warnings) factor. Third, the involvement of multiple parties and the lack of clearly allocated responsibilities among the parties contributed to the failure, implicating *fragmentation* and *organizational characteristics* (responsibility division) in the HOPE framework. Lastly, the DSB pointed to the absence of a collective safety approach and insufficient failure risk assessment concerning the supporting structure, which corresponds to the *engineering climate* and *risk analysis and management* factors within the HOPE framework.

#### 3.3.2 FC Twente Stadium

The extended roof structure of the FC Twente stadium collapsed on July 7, 2011, claiming two lives and leaving nine injured. The roof collapse was initiated by the failure of a roof beam. Due to time constraints, the roof construction process was changed from sequential to simultaneous, leading the beam to be overloaded before it was sufficiently stabilized. While the direct failure cause differs, this failure shares some similar underlying causes as the Rotterdam B‐Tower case, such as a lack of a joint safety approach between parties, unclear responsibility allocation, and inadequate checking and supervision. Additionally, this case exposed other latent factors. The DSB pointed out that decisions associated with structural safety were not made at the appropriate organizational level. This is associated with the *organizational characteristics* factor in the HOPE framework. Moreover, the investigation disclosed that the parties collaborated based on mutual trust in each other's professional competence without verifying the required prerequisites before conducting the next steps. This trust is based on the past collaboration experience between the parties. This underlying factor is closely linked to the factor of *trust* (reliance on other parties, over‐confident about past experience, blind trust without verification) in the HOPE framework. Even though *task management* is not recognized as a critical factor in this survey study, the FC Twente case revealed its importance in structural safety, especially change management as well as task coordination and collaboration, as listed in the HOPE framework.

#### 3.3.3 Eindhoven Airport Parking Building

On May 27, 2017, the Eindhoven Airport parking building partially collapsed. Fortunately, no causality or injury was caused. The DSB recognized the direct cause of this failure as the wrong design decision to rotate the floor slabs in their installation while failing to anticipate or assess the potential consequences of this design change. In the end, the longitudinal shear capacity between prefab and cast‐in‐situ concrete at the floor slab seams was insufficient. While investigating the underlying conditions that contributed to this failure, the DSB arrived at conclusions that are strikingly similar to the findings of the previous two structural failure investigations. The Eindhoven case was also subject to the lack of a clear responsibility distribution and collective attention toward structural safety. In addition, the DSB identified the existence of a detrimental blame culture in the Dutch construction sector. This is related to the *engineering climate* (safety culture) factor in the HOPE framework. The DSB proposed the elimination of this blame culture to foster a culture of learning from past incidents, thereby facilitating continuous improvement in structural safety. Furthermore, the factors of *fragmentation* and *organizational characteristics* (complex project organization structure) were spotted as contributing underlying conditions to this structural failure. Most importantly, the DSB highlighted the crucial role played by a strong focus on the lowest price in limiting the allocation of adequate resources and attention to risk. This is reflected in the *stress* (budget pressure) factor outlined in the HOPE framework.

## 4 Discussion

The proposed HOPE framework can assist project managers and engineers in gaining an overall vision of the safety of the structure taking into consideration the subtle, often invisible, yet rather critical impacts from the “soft” human and organizational aspects of the project system. This deliberation is largely missing in engineering practice. Therefore, with the help of the HOPE framework, the potential human and managerial hazards that threaten structural safety can be identified proactively. Additionally, the HOPE framework can be used as a tool to deliver structural quality assurance support, with which better allocation of structural safety management resources can be achieved.

With the obtained results from this survey study, the critical HOFs that contribute to the occurrence of human errors in each GTT in the structural design and construction process have been identified through expert judgments for the Dutch construction industry. These results can assist professionals with more specific human error‐oriented risk identification and management in practice. Additionally, quality assurance resources should be leaned toward the vulnerable task types when considering their error proneness. Furthermore, these results lay the groundwork for the future development of a tailored HRA method for the construction industry, which is currently absent. HRA has been an essential component of an overall Quantitative Risk Analysis (QRA) for a system in many safety‐critical industries such as nuclear, aviation, and chemical processing. Therefore, developing an HRA method for the construction industry can complete the long‐ignored human contribution puzzle in the structural failure risk analysis. In the following subsection, the identified HOFs are cross‐validated with additional sources of study findings.

### 4.1 Compare HOFs With PSFs

The identified subcategory HOFs are further compared with the PSFs/PIFs in widely applied HRA methods and studies, including INTENT [39], HRMS [40], CREAM [35], SPAR‐H [41], Good Practices for HRA [42], and the PIF hierarchy proposed by Groth and Mosleh [43]. The outcome of this comparison is presented in Table 4. The comparative analysis reveals that the majority of the identified subcategory HOFs are covered in these reviewed methods and studies through one or several specific HOFs as outlined in the HOPE framework. This alignment indicates a broad consensus on the underlying conditions contributing to human error occurrences across industries.

**Table 4:** Comparison of the identified HOFs for the Dutch construction industry with PSFs from existing HRA methods.

HOFs for Dutch construction	INTENT	HRMS	CREAM	SPAR‐H	Good practices for HRA	PIF hierarchy
Professional competence	Experience, training	Training/expertise/experience/competence	Adequacy of training and experience	Experience/training	Training/experience	Knowledge/experience, skills, familiarity with situation
Trust						
Attitude	Motivation					Bias, morale/motivation/attitude
Well‐being for duty				Fitness for duty	Special fitness needs	Physical/psychological abilities
Comprehensive abilities					Crew characteristics	Role awareness, attention
Information flow	Communication	Quality of information			Communication	Necessary information, communication
Organizational characteristics						
Quality assurance	Supervision, procedures	Procedures	Availability of procedures	Procedures	Suitability of relevant procedures and administrative controls	Training program, corrective action program, procedures, direct supervision
Engineering climate	Safety culture					Safety culture
Complexity		Task complexity	Number of simultaneous goals	Complexity	Complexity	Task complexity
Stress	Stress, workload	Time	Available time	Stress/stressors, available time	Time availability, workload/time pressure/stress, available staffing/resources	Staffing/scheduling, task/time load, stress
Fragmentation						
Equipment	HMI	Quality of interface	Adequacy of MMI and operational support	Ergonomics/HMI	Availability and clarity of instrumentation, ergonomic quality of human‐system interface (HSI), accessibility and operability of the equipment, need for special tools	Tools, HIS, system response
Working conditions			Working conditions, time of day		Environment	Workplace adequacy, external environment
Task management		Task organization	Adequacy of organization, availability of plans, crew collaboration quality	Work process	Team/crew dynamics	Team coordination/cohesion
Risk analysis and management					Consideration of realistic accident sequence diversions and deviations	Perceived situation, perceived decision

However, differences between the critical HOFs and PSFs reveal intriguing insights. Specifically, the factors of *trust*, *organizational characteristics*, and *fragmentation* are recognized as critical HOFs in the Dutch construction industry but are not encompassed within any of the reviewed HRA methods and studies. Consequently, these three HOFs can be regarded as unique error‐inducing factors specific to the Dutch construction industry, a finding confirmed by the analysis presented in Section 3.3, where these factors were frequently identified as critical underlying contributors to structural failures in the Netherlands.

Moreover, the factors of *task management* and *risk analysis and management* do not attain the status of critical HOFs for the Dutch construction industry in this survey, despite their inclusion as latent factors contributing to human errors in many of the reviewed HRA methods and studies. It is important to clarify that their omission from the list of critical HOFs in this study should not be interpreted as implying their negligible influence on human error occurrences. Rather, this outcome suggests that these two factors are relatively less significant when compared to the other 14 HOFs under consideration.

### 4.2 The Application of the Critical HOFs and the HOPE Framework

Given the global scope of the reviewed literature, the synthesized HOPE framework is considered to be applicable to the broader construction industry worldwide. However, it is important to note that the critical HOFs, a more selective subset of factors from the HOPE framework and identified through a survey involving experts from the Dutch construction sector, exhibit a greater specificity to the circumstances within the Dutch construction industry. Consequently, these critical HOFs cannot be generalized to the construction industries of other nations without undergoing further investigation and adaptation.

When to consult the HOPE framework and when to focus on the critical HOFs? The choice hinges upon the specific objective of the analysis. When the analysis seeks to provide qualitative insights into various underlying conditions that lead to human errors or pinpoint potential structural failure risks, the comprehensive array of specific HOFs outlined at the bottom hierarchy of the HOPE framework is better suited for this purpose. On the other hand, when the goal is to assess human error likelihood and the associated structural failure risks, then the critical HOFs offer practical risk assessment by focusing only on the factors with significant impacts [44].

## 5 Conclusions

A primary contribution of this study lies in the introduction of the HOPE framework, a comprehensive, hierarchical taxonomy of latent factors behind human errors. This framework serves as an insightful guide for practitioners in the construction industry, facilitating improved treatment of human errors and identification of structural failure risks. It encompasses considerations related to human factors, organizational factors, project factors, and environmental factors. Drawing upon this framework, a survey study was conducted to pinpoint critical HOFs that exert significant influence on human error occurrence in structural design and construction tasks. Findings from this survey yield an enhanced understanding of task‐specific underlying conditions contributing to human errors within the Dutch construction sector. This knowledge can be instrumental in aiding professionals in implementing more effective quality assurance measures for structural safety. In addition, the critical HOFs identified for each GTT, as shown in Figure 6, lay the foundation for the future development of a quantitative HRA method tailored for the Dutch construction industry.

## Author Contributions

**Xin Ren:** writing–original draft, investigation, funding acquisition, formal analysis, data curation. **Karel C. Terwel:** writing–review and editing, supervision, conceptualization. **Ming Yang:** writing–review and editing, supervision, methodology. **Pieter H. A. J. M. van Gelder:** writing–review and editing, supervision, methodology, conceptualization.

## Consent

Consent was obtained from all participants in the study after presentation of written materials describing the procedures and survey.

## Conflicts of Interest

The authors declare no conflicts of interest.

## Disclosure by AJIM Editor of Record

John Meyer declares that he has no conflicts of interest in the review and publication decision regarding this article.

## Disclaimer

The authors declare that they have no known competing financial interests or personal relationships that could have appeared to influence the work reported in this paper.

## Supporting information

Supporting information.

## Data Availability

The data that support the findings of this study are available from the corresponding author upon reasonable request.
