# Supplementary material for: Critical Human and Organizational Factors for Structural Safety in the Dutch Construction Industry
Source: Am J Ind Med. 2024 Dec 3;68(Suppl 1):S115–30. doi: 10.1002/ajim.23681 (PMC11869801; doi:10.1002/ajim.23681)
Supplement: Supplementary file 1 — Supporting information. [file AJIM-68-S115-s001.docx]

*
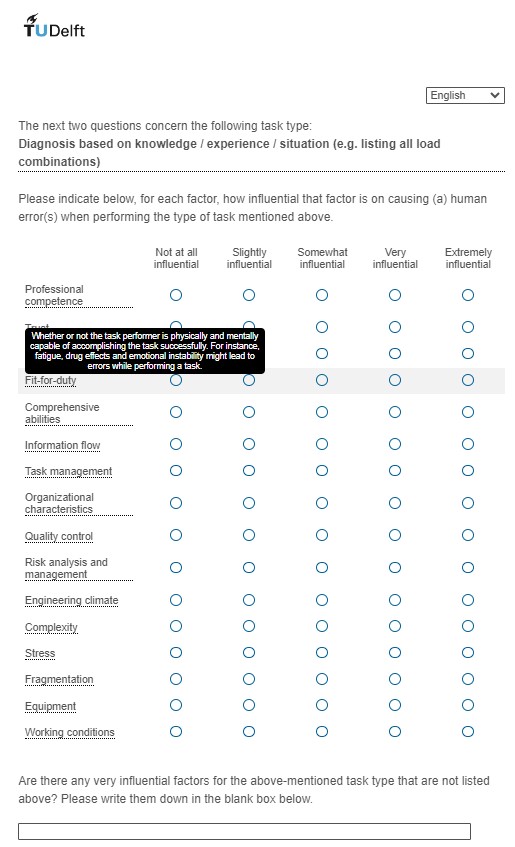
*

**FIGURE S-1** Survey question example*.*

The question description furnishes both the definition of the queried GTT and an example task. Additionally, the definition of each factor is accessible to the respondent through an information box, which becomes visible when the cursor hovers over the respective factor label. The inclusion of this reference serves the purpose of enhancing the reliability of the collected data by establishing a shared understanding of the meaning of each factor and the aspects to be considered when providing the rating.
